# Underwater Turbid Media Stokes-Based Polarimetric Recovery

**DOI:** 10.3390/s24051367

**Published:** 2024-02-20

**Authors:** Zhenfei Wang, Meixin Hu, Ketao Zhang

**Affiliations:** 1 Centre for Advanced Robotics at Queen Mary (ARQ), School of Engineering and Materials Science, Queen Mary University of London, Mile End Road, London E1 4NS, UK; ketao.zhang@qmul.ac.uk; 2 School of Mathematics and Physics, North China Electric Power University, Beijing 102206, China; 120222209001@ncepu.edu.cn

**Keywords:** polarization, underwater imaging, polarimetric imaging, turbid media, optical sensors, optical measurements

## Abstract

Underwater optical imaging for information acquisition has always been an innovative and crucial research direction. Unlike imaging in the air medium, the underwater optical environment is more intricate. From an optical perspective, natural factors such as turbulence and suspended particles in the water cause issues like light scattering and attenuation, leading to color distortion, loss of details, decreased contrast, and overall blurriness. These challenges significantly impact the acquisition of underwater image information, rendering subsequent algorithms reliant on such data unable to function properly. Therefore, this paper proposes a method for underwater image restoration using Stokes linearly polarized light, specifically tailored to the challenges of underwater complex optical imaging environments. This method effectively utilizes linear polarization information and designs a system that uses the information of the first few frames to calculate the enhanced images of the later frames. By doing so, it achieves real-time underwater Stokes linear polarized imaging while minimizing human interference during the imaging process. Furthermore, the paper provides a comprehensive analysis of the deficiencies observed during the testing of the method and proposes improvement perspectives, along with offering insights into potential future research directions.

## 1. Introduction

High-definition underwater optical imaging has always been a challenging and highly valuable research topic, providing significant technical support for various underwater machine operations, such as underwater target recognition, environmental detection, and fish population management. Polarized light imaging is a method that utilizes multiple angle-polarized images to recover clear images. This approach leverages one of the fundamental properties of light polarization, where light vibrates perpendicular to its direction of propagation. A polarizing filter is an optical instrument that restricts the passage of light at certain angles. It allows light with vibrations parallel to the narrow slit to pass through while blocking light with vibrations perpendicular to it. When light propagates in water, it is subject to absorption and scattering due to the density and absorption effects of solutes in the water [1]. In this scenario, a polarized light sensor can effectively filter out stray light produced during these processes, allowing for a better representation of the detailed information in images. In traditional polarized light sensors, specific angle polarizers are typically set in front of the imaging device to obtain polarized light imaging results at a certain angle. In this mode, multiple sets of polarized light sensors are often needed simultaneously to meet practical requirements. The operation of multiple sets of polarized sensors usually requires a significant budget and results in a larger volume, hindering widespread application. However, the emergence of the Sony IMX250 series polarized light sensor has changed this situation. Each pixel in this sensor has its own polarizing filter. This polarized light sensor has a unique structure, with each pixel’s polarizing filter coated with an anti-reflective layer and positioned between micro-lenses and photodiodes [2]. Therefore, it can capture polarized light photos containing information from four different angles in a single shot. The miniaturization of this polarized light sensor can effectively facilitate the application of polarized light technology in underwater robotics.

There are three classical methods of polarized light imaging. The first is Polarization Difference Imaging (PD), which involves using two orthogonal polarized images at different angles to estimate transmittance and obtain a restored clear image [3]. For instance, Rowe and Tyo utilized PD as a common-mode suppressor to mitigate the influence of backward scattered light, amplifying the PD amplitude from the background to detect details that are not easily discernible, providing a novel approach to underwater imaging [4]. In 1996, Tyo and Rowe studied the restoration capability of PD and the polarization sum under different scattering conditions, and PD was found to significantly enhance the target detection range [3]. Schechner proposed a method that establishes an underwater imaging model by solving the backscatter PD using two orthogonal polarized images and referencing the atmospheric imaging model. This fusion enables the application of polarized imaging in underwater environments [5]. The second method is Stokes Polarization Imaging (SP), which utilizes the robustness of polarization angles to eliminate backscattered light and achieve image clarity [6]. For instance, Tian et al. combined occluded light Stokes vectors with differential polarization imaging, devising an improved PD imaging method. The main advantage of this approach is its ability to rapidly restore images through algorithmic calculations, given the Stokes parameters, providing PD imaging with fast imaging capabilities [7]. In summary, as Stokes vectors offer additional valuable information in images, such as Degree of Polarization (DoP) and Angle of Polarization (AoP), which are typically related to backscatter caused by water particles and background light, methods based on Stokes polarization imaging generally outperform traditional polarization imaging techniques in image restoration [8]. In 2009, Setälä et al. demonstrated the disparity between DoP in the time and frequency domains. In this article, they employed mathematical derivations to establish that the spectral degree of polarization is contingent upon the temporal coherence of the field, whereas the temporal degree of polarization is not necessarily so [9]. This mathematical foundation enhances the theoretical underpinning for the improved application of DoP. Thus, increasing information dimensions and optimizing parameters can effectively enhance the imaging quality. The third method is Mueller Matrix Imaging (MM), which can better utilize all the information of polarized light, resulting in improved polarization image restoration [10]. Initially, polarization imaging of scattering media based on MM involved linear polarization imaging with the modulation of active illumination, which typically required nine images for computation [11]. Wang et al. analyzed the physical optical model of backward scattered light and target reflected light, altering the angle between the polarizer direction and the direction of backscattered light. By combining MM imaging with PD imaging, they successfully eliminated scattered light, achieving excellent output results under ideal conditions [12]. Compared to the first two polarization imaging methods, MM-based approaches have the advantage of utilizing more useful image information and increasing polarization degrees of freedom, but they also require higher data processing and computational complexity. Overall, each polarized imaging method has its strengths and weaknesses, making it meaningful to choose the appropriate approach for polarization imaging based on different application scenarios.

We recognize the significance of real-time imaging techniques in various underwater machine applications. Algorithms capable of real-time imaging are more suitable for practical underwater working environments compared to those limited to image processing only [13]. They can often reveal more details present in the underwater environment, such as detecting cracks in underwater structures or salvaging underwater objects, tasks that require prolonged and effective detection by underwater machinery. Traditional underwater polarized light imaging algorithms, however, are often limited to processing a single or a few images [14], lacking the ability to process video data over an extended period. This limitation greatly hinders the application of various polarized imaging techniques in underwater environments. To address this drawback, we propose a real-time underwater image dehazing technique based on Stokes polarized imaging. This method capitalizes on the slow movement speed of underwater robots, ensuring that environmental light intensity and underwater conditions do not change significantly over short periods. The approach involves using the information from the first frame to dehaze subsequent images. It first processes the first frame, calculates the background light of this frame, and then uses the estimated background light to dehaze subsequent images. Simultaneously, it computes the clarity metrics of these images, and if they do not meet the standard, it recalculates the background light and repeats the process. This method proves effective in scenarios where parameters affecting background light estimation, such as underwater light intensity, solute–solvent interactions, and impurities, change little over short periods in underwater slow-motion robots. To validate the effectiveness of the proposed method, we conducted several practical experiments in turbid water. Finally, we acknowledge the limitations and shortcomings of this method and provide its recommendations for improvement. This research also offers some perspectives on underwater polarized light modeling.

This paper is structured as follows: In Section 2, we provide the classical principles of underwater polarized light imaging and the innovative contributions made in this work. In Section 3, we elaborate on the experimental conditions and outcomes, providing a detailed account of the experimental setup and results. In Section 4, we present conclusions and future prospects.

## 2. Methodology of Imaging Recovery by Stokes Polarization in Real-Time Imaging

### 2.1. Traditional Underwater Polarization Imaging Model

**Figure 1:**
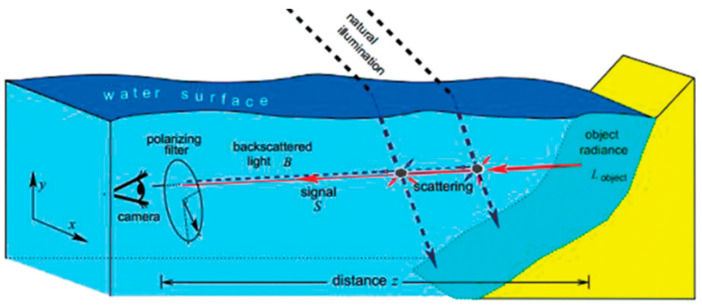
Physical model of underwater passive polarization imaging [5].

In polarized imaging models, based on the light source used, polarized imaging can be categorized into active polarized imaging and passive polarized imaging. Active polarized imaging refers to polarized imaging with an active polarized light source, primarily aiming to eliminate the absorption, scattering, and attenuation of water on natural light. Traditional passive polarized imaging, on the other hand, does not require an artificial light source and mainly relies on natural light. However, as research advances, an increasing number of passive polarized imaging systems have also started to introduce polarized light sources to counter water interference. In 2005, Schechner et al. proposed the classic model for uniform turbid media underwater polarized imaging, as shown in Figure 1 [5].

This model posits that the polarization degree of the target information light is much smaller than that of the background scattering, allowing us to neglect forward scattering. Based on this premise, the total received light by the camera can be divided into the direct transmitted light $D\left(x,\;y\right)$ radiated by the target object and the background light $B\left(x,\;y\right)$ formed by the scattering particles in the water. $L\left(x,\;y\right)$ represents the brightness of the object that is neither scattered nor absorbed by the water particles, typically used to denote the recovered image. The transmission coefficient of the scattering medium, $t\left(x,\;y\right)$, generally varies with changes in the underwater environment, while $A_{\infty }$ denotes the background scattering light at an infinite distance, which also varies with the intensity of light and the underwater conditions. The mathematical formulation of this model is given by the following Equation (1).

(1) $$I\left(x,\;y\right)=D\left(x,\;y\right)+B\left(x,\;y\right),\;I\left(x,\;y\right)=L\left(x,\;y\right)\cdot t\left(x,\;y\right)+A_{\infty }\left[1\;-\;t\left(x,\;y\right)\right].$$

Traditional polarization imaging restoration methods are based on two orthogonal polarized images, namely the co-linear image and the cross-linear image [15]. These two polarized images can be represented by the following Equation (2).

(2) $$I^{\|}\left(x,\;y\right)=\frac{D(x,\;y)}{2}+B^{\|}\left(x,\;y\right),\;I^{⊥}\left(x,\;y\right)=\frac{D(x,\;y)}{2}+B^{⊥}\left(x,\;y\right).$$

Hence, the backscattered DoP can be represented as $P_{\mathrm{scat}}=(A_{\infty }^{\|}-A_{\infty }^{⊥})/(A_{\infty \;}^{\|}+A_{\infty }^{⊥})$, and the veiling light can be expressed as $B(x,\;y)=(I^{\|}-I^{⊥})/\;P_{\mathrm{scat}}$ [16]. Subsequently, the transmittance and scene brightness can be obtained through calculations as shown in Equation (3).

(3) $$t(x,\;y)=1-\frac{I^{\|}(x,\;y)\;-I^{⊥}\left(x,\;y\right)}{P_{\mathrm{scat}}A_{\infty }},\;L(x,\;y)=\frac{I(x,\;y)\;-B(x,\;y)}{t(x,\;y)}=\frac{I^{\|}(x,\;y)+I^{⊥}(x,\;y)\;-B(x,\;y)}{t(x,\;y)}.$$

### 2.2. Traditional Stokes Polarization Parameter Method

The previously mentioned model describes Schechner’s traditional polarization image recovery based on linearly polarized light. Many current research models and innovations build upon this foundation to enhance and update the approach. Moreover, the calculations for $I^{\|}$ and $I^{⊥}$ can be replaced and computed using other parameters, allowing the model to be adapted for different scenarios. In comparison, Stokes polarimetry outperforms traditional polarization imaging as it better utilizes DoP and AoP. In traditional polarization imaging, target light is always partially polarized, making it challenging to obtain an ideal polarization sub-image with a rotating polarizer [17]. Therefore, Stokes polarimetry can more effectively recover image information. The Stokes vector [I Q U V] describes both the intensity and polarization (including DoP and AoP) information of the light wave. However, obtaining the Stokes vector using previous methods has been complex and computationally challenging [18]. The radiation received by the detector includes linearly and circularly polarized light. Thus, the Stokes vector can be specifically divided into linearly polarized, circularly polarized, and unpolarized components [19]. The representation is shown in Equation (4), where the table from left to right represents linearly polarized light, circularly polarized light, and the unpolarized component. The DoP is a physical quantity that characterizes the polarization degree of an electromagnetic wave. It is a value between 0 and 1, representing the proportion of polarization in the total power of an electromagnetic wave. $P_{polarized}$ is the power of the polarized component, and $P_{total}$ is the total power. The AoP is the angle between the vibration direction of the electromagnetic wave and a reference direction. When polarization occurs in the electromagnetic wave, the angle between the vibration direction and the direction of wave propagation is the polarization angle. These two physical quantities play a crucial role in describing polarized light. The process for calculating AoP and DoP is also provided in Equation (4).

(4) $$S=S_{l-\mathrm{polarized}}+{\;S}_{c-\mathrm{polarized}}+S_{\mathrm{unpolarized}},\;S=\left[\begin{array}{c} \begin{array}{c} \sqrt{S_{1}^{2}+S_{2}^{2}}\\ S_{1} \end{array}\\ \begin{array}{c} S_{2}\\ 0 \end{array} \end{array}\right]+\left[\begin{array}{c} \begin{array}{c} S_{3}\\ 0 \end{array}\\ \begin{array}{c} 0\\ S_{3} \end{array} \end{array}\right]+\left[\begin{array}{c} \begin{array}{c} S_{0}-\sqrt{S_{1}^{2}+S_{2}^{2}}-S_{3}\\ 0 \end{array}\\ \begin{array}{c} 0\\ 0 \end{array} \end{array}\right],\;\mathrm{AoP}=\frac{1}{2}\tan^{-1}\left[\frac{S_{2}}{S_{1}}\right],\;\mathrm{DoP}=\frac{P_{polarized}}{P_{total}}=\frac{\sqrt{S_{1}^{2}+S_{2}^{2}+S_{3}^{2}}}{S_{0}}.$$

Indeed, calculating Stokes polarization parameters can be a complex process, involving considerations of the polarization distribution of Stokes vectors. The specific derivation process can be quite intricate, and detailed explanations have been provided in research articles. For a comprehensive understanding of the derivation process, you may refer to the work by Wei and colleagues, which presents a novel method for utilizing Stokes polarization to enhance underwater image quality [20]. It is important to note that the Stokes polarization parameters offer valuable information about the polarization state of light and can be highly beneficial in various applications, including underwater imaging. Researchers have continuously worked on refining and improving methods for deriving and utilizing Stokes parameters to better extract and enhance information from polarized light in underwater environments.

### 2.3. Stokes-Based Underwater Polarization Imaging Method

In 2014, Liang et al. conducted research on the relationship between Stokes vectors and veil light, building on previous studies related to the expression of polarized light using Stokes vectors [21]. They proposed a method to estimate the backward scattering/airlight based on the analysis of AoP. This approach involved capturing images at different polarization angles, 0°, 45°, and 90°, which are demonstrated with reference in Section 3. The formula for solving the linear polarized Stokes parameters that they proposed is shown in Equation (5).

(5) $$S_{0}=I\left(0\right)+I\left(90\right),\;S_{1}=I\left(0\right)-I\left(90\right),\;S_{2}=2I\left(45\right)-S_{0}.$$

This method optimizes the process of solving the Stokes vector and greatly enhances the system’s robustness while reducing sensitivity to noise. In real-world machine working scenarios, circularly polarized light is more challenging to acquire, as it requires higher demands on angles and generation conditions. On the other hand, linearly polarized light is easier to generate and detect. Therefore, in this paper, we choose to use the linear polarized Stokes parameters, as represented by Equation (5), for image restoration, integrating these parameters into the underwater physical model. In the proposed Stokes underwater linear polarized imaging model, we first need to process the linearly polarized light as described in a method similar to what was mentioned in Section 2.1. We need to obtain the background scattering light ($A_{\infty }$) and the linear polarization degree ($P_{\mathrm{scat}}$) at infinity. They are presented by the following Equation (6), where Ω represents the selected background region, specifically the number of pixels within the background area [22].

(6) $$A_{\infty }=\frac{1}{\left|\Omega \right|}\sum_{(x,\;y)\in \Omega }S_{0}\left(x,\;y\right),\;P_{\mathrm{scat}}=\frac{1}{\left|\Omega \right|}\sum_{(x,\;y)\in \Omega }\left[\frac{\sqrt{{\left[S_{1}\left(x,\;y\right)\right]}^{2}+{\left[S_{2}\left(x,\;y\right)\right]}^{2}}}{S_{0}\left(x,\;y\right)}\right].$$

In practical estimation processes, slight variations in the above two parameters may occur due to different background light selections. To address this, we can introduce an additional parameter, denoted as $\varepsilon_{l}$, to constrain $P_{\mathrm{scat}}$ as $\varepsilon_{l}P_{\mathrm{scat}}$. This parameter can be designed as a fixed value for a specific underwater scenario or set as a constrained index through multiple optimizations to determine the best solution. While the fixed value approach is simpler, the optimization-based method yields more accurate results at the cost of longer processing times. If computational resources are abundant, I recommend using the second approach, as it can lead to clearer imaging results. By substituting Equation (6) into the process of solving the medium transmittance and object radiance, as shown in Equation (3), we can derive the Stokes-based linear polarized underwater physical imaging model, as presented in Equation (7).

(7) $$t(x,\;y)=1-\frac{\sqrt{{\left[S_{1}\left(x,\;y\right)\right]}^{2}+{\;\left[S_{2}\left(x,\;y\right)\right]}^{2}}}{\varepsilon_{l}P_{\mathrm{scat}}A_{\infty }},\;L(x,\;y)=\frac{I(x,\;y)\;-B(x,\;y)}{t(x,\;y)}=\frac{S_{0}(x,\;y)\;-A_{\infty }\left[1\;-t(x,\;y)\right]}{t(x,\;y)}.$$

Based on the equations mentioned earlier, we can develop a waterborne image restoration method by combining Equations (5) and (6). This method utilizes the simplified equation proposed by Liang et al. for solving Stokes vector parameters and integrates it with the traditional underwater linear polarized model, resulting in a novel Stokes-based linear polarized underwater physical imaging model. Additionally, to address the requirements of underwater robotics in processing videos, a system for underwater video enhancement has been designed to minimize human intervention.

### 2.4. Underwater Video Processing Method Based on Stokes Linear Polarization

**Figure 2:**
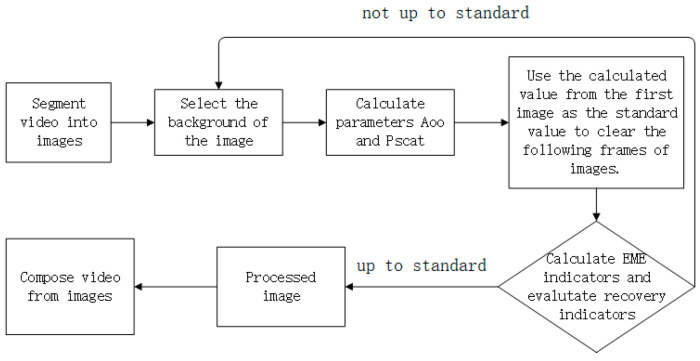
Algorithm flowchart.

We delve into the current state of underwater polarized imaging research, revealing that the existing processing methods and algorithms mainly focus on enhancing images. Their primary aim is to optimize the computation process and incorporate more image information to achieve better clarity and restore additional image features in underwater polarized images. While these studies progress, they often contribute to refining the fundamental theories of underwater polarized imaging and explore the integration of other image processing algorithms to achieve superior results [23]. Considering the operational environment of underwater robots, it becomes evident that applying this technology to underwater robotics faces challenges due to the size constraints of underwater imaging platforms. Most underwater robots worldwide are striving for miniaturization, which makes processing individual photos with polarized imaging technology costly and inefficient. In underwater robotics applications, real-time video imaging capabilities are typically required. Therefore, we design a method for processing video sequences underwater, aiming to minimize human intervention and reduce labor efforts. The main process of this method is depicted in Figure 2.

To address the classical underwater polarized imaging methods that require selecting background light regions for estimating background light intensity and possibly estimating the parameters in Equation (7), the proposed approach leverages the stable underwater environment, where solute concentration and light intensity change insignificantly over short periods. By using the extracted $A_{\infty }$ and $P_{\mathrm{scat}}$ from the first frame of the video sequence as indicators for subsequent frames, human intervention is reduced. Firstly, we segment the captured video into a series of images, one for each frame. For the initial frame, we manually designate the background light region and use Equation (6) to compute the average light intensity parameters ($A_{\infty }$) and linear polarization degree ($P_{\mathrm{scat}}$) for that region. Secondly, we decompose the captured images arranged by channels to obtain polarized images in four different directions: 0°, 45°, 90° and 135°, as shown in Section 3. Employing Equation (5), we calculate $S_{0}$, $S_{1}$, and $S_{2}$. Taking these three Stokes parameters along with environmental parameters $A_{\infty }$ and $P_{\mathrm{scat}}$, obtained from the initial frame, we input them into Equation (7) to calculate the enhanced result for the initial frame. Thirdly, using the environmental parameters obtained from the initial frame and $S_{0}$, $S_{1}$, and $S_{2}$ calculated from the second frame using Equation (5), we compute the enhanced result for the second frame using Equation (7). Fourthly, after enhancing the second frame, we assess the enhancement effect using image evaluation metrics. If the evaluation meets the predefined criteria, the enhancement result for the second frame is considered acceptable. If the evaluation criteria are not met, we manually select the background light for the second frame, recalculate the environmental parameters, and then apply them to image enhancement. This process is repeated until the end of the image sequence. Regarding the selection of evaluation metrics, a set of photos can be pre-captured underwater and enhanced. Observing this set of photos and determining the satisfactory response results will provide a reference value for evaluation. This reference value can then be chosen as the standard for assessment. Finally, we concatenate the processed image sequence to obtain the processed video. This approach effectively reduces the manual effort required for polarized light processing in a long video, while ensuring a certain level of accuracy in the image results. Additionally, the Enhancement Measure Evaluation (EME) metric is employed to assess the restoration results. EME quantifies the local grey-level variations in the image; stronger variations indicate more pronounced image details. Its calculation is based on segmenting the image and computing the logarithmic mean of the ratio between the maximum and minimum grey values within the segmented regions. For blurry underwater images, EME proves to be an effective indicator of image enhancement quality.

## 3. Experiments and Results

In this section, we simulate real underwater imaging experiments in a laboratory pool to validate the underwater imaging model and the algorithm proposed in the second Section 2. The process involved capturing various images and videos within the simulated pool environment and subsequently applying algorithms to process these visuals, thereby substantiating the feasibility of the methods presented in the Section 2. By introducing varying volumes of milk into the water pool, the experiment emulated different levels of underwater turbidity. Altering the milk volume facilitated the simulation of distinct degrees of water turbidity [24]. Milk, being a complex substance composed of water, fats, proteins, lactose, and particles of varying sizes, is capable of modulating the scattering characteristics of a solution due to the diverse optical properties of its constituents. Hence, milk serves as an effective liquid for simulating underwater polarized environments.

**Figure 3:**
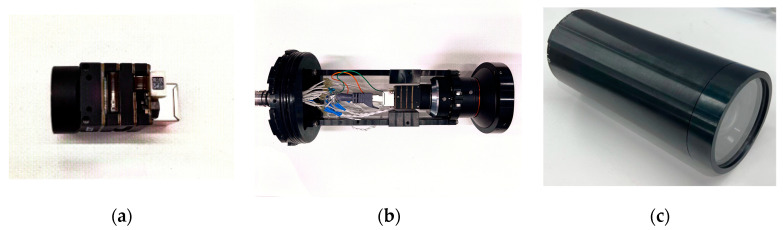
(**a**) LUCID PHX050S-QC color polarization camera; (**b**) camera encapsulated inside the cylindrical metal waterproof; (**c**) camera final encapsulation.

In this experiment, we designed and utilized a novel image sensor measurement system. This system comprises a polarization camera (LUCID PHX050S-QC color polarization camera) and a fixed focal length lens for acquiring polarized images. The setup is fixed within a cylindrical metal waterproof housing, as depicted in Figure 3c.

**Figure 4:**
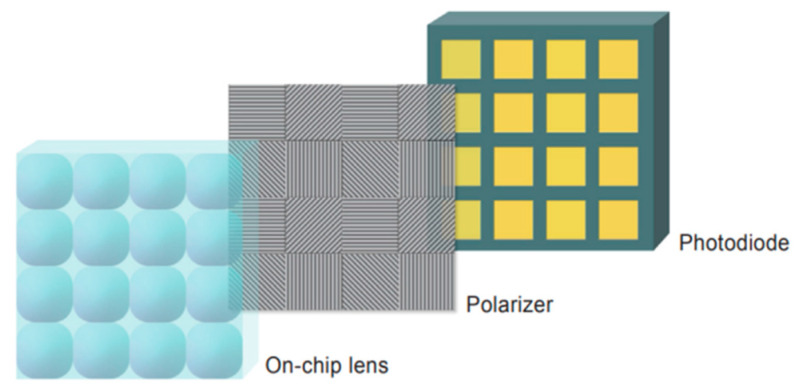
Polarization sensor structure decomposition [2]. It has a three-layer structure with a lens, a polarizer, and a photodiode in that order.

The camera employs a CMOS-based image sensor (Sony IMX250MYR polarization sensor). This sensor is equipped with a layer of linear micro-polarizers in different directions on its photodiodes, as illustrated in Figure 4. This design enables it to simultaneously capture images with four different polarization directions, and it reduces cost and interference compared to acquiring images from four angles by adjusting the polarizer angle and repeating the shot. The camera boasts a resolution of 5 million pixels.

**Figure 5:**
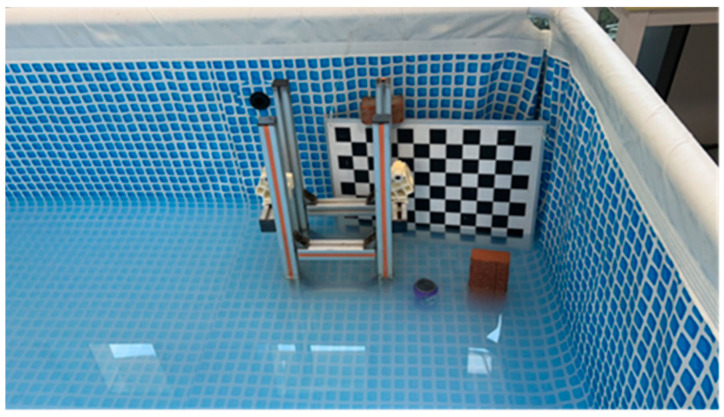
Target objects in the pool.

In the course of experiments, four distinct objects were chosen for target recognition. In order to comprehensively represent scenarios that an underwater robot might encounter, cracked bricks were employed to emulate stone materials typically found in underwater structures, submerged steel frame structures simulated metallic materials, beverage cans bearing text and images represented scenarios involving underwater text and image observation, while a checkerboard pattern was introduced to enhance image recovery contrast. The experimentation involved two distinct scenarios: images and videos, with processes that shared some similarities. Ensuring controlled variables, the study maintained stable external lighting conditions and conducted steady captures at a distance of 10 cm from the objects. This approach yielded imaging outcomes under varying concentrations. Simultaneously, maintaining the same distance, the camera was slowly moved in parallel to obtain video recordings of the four objects under different concentrations. To offer readers a better understanding of the experimental setup, Figure 5 below illustrates an overhead view of the testing pool containing the four designated test objects.

**Figure 6:**
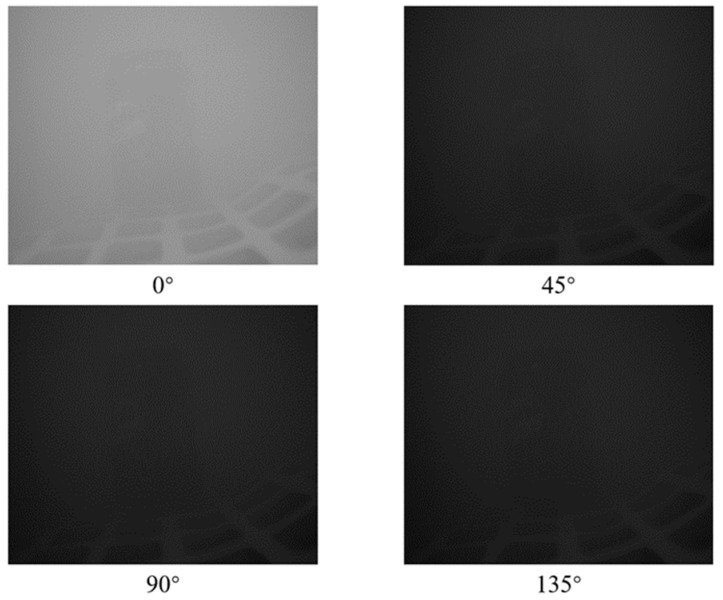
Polarized images in four directions. These angles represent the angle of the polarizer, which allows polarized light parallel to that angle to pass through.

To derive the Stokes parameters, we initially dissect the polarized images acquired by the polarized camera into four separate images representing the four polarization directions. Figure 6 presents the images captured with the polarized camera and the four images obtained after decomposition. These four images represent the imaging results of the original underwater light under the action of four polarizers with different angles. Following the computational approach outlined by Equation (5) in the article, the four parameters of the Stokes vector can be calculated.

Upon obtaining the Stokes vector, the paper proceeds to estimate background scattered light and polarization degree by selecting the appropriate background illumination. By incorporating a constraint parameter $\varepsilon_{l}$, the computational process yields enhanced image results. For the video dataset, a constraint is enforced based on image enhancement evaluation metrics. If the restored image result does not meet the evaluation criteria, a reiteration of the background light parameter calculation ensues and is applied to subsequent image enhancements.

**Figure 7:**
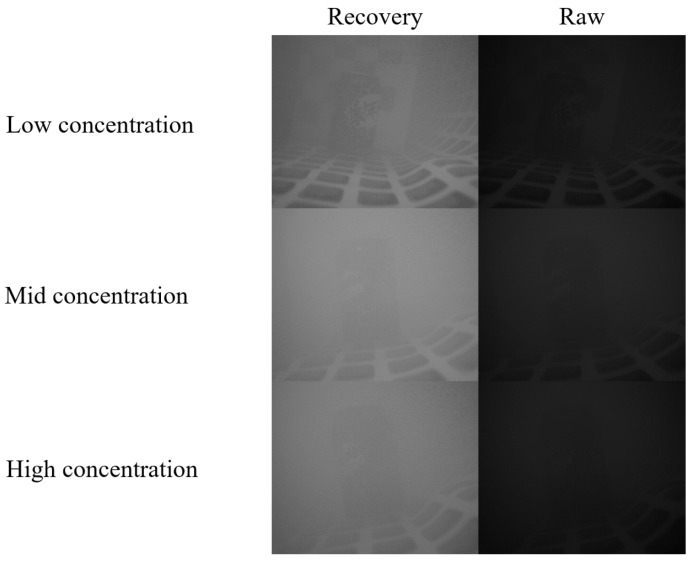
Image enhancement results at three concentrations. Low concentration is 200 mL of milk added to the pool. Medium concentration is 400 mL of milk added to the pool. High concentration is 600 mL of milk added to the pool. The volume of water in the pool is about 200 L.

We executed Python programs on a personal laptop featuring an AMD Ryzen 7 5800H CPU@ 3.20 GHz processor. The choice of $\varepsilon_{l}$ can be based on specific camera parameters in the given environment and can be determined by selecting reference empirical values under different operational conditions. Alternatively, the optimal solution can be obtained through multiple practical experiments. The paper conducted image enhancement on photos depicting three distinct turbidity levels. To visually demonstrate the image restoration effects more intuitively, Figure 7 compares the results of image enhancement with the original photos before enhancement. In Figure 7, we present three sets of image enhancement results under different solution turbidity conditions, namely low concentration, medium concentration, and high concentration. It can be observed that under low concentration conditions, the restoration effect on the checkerboard pattern and text is most pronounced. Under medium to high concentration conditions, there is a more noticeable enhancement effect on the images and text contours on the can body. It is noteworthy that the camera used captures four different angle images in a single shot, rendering the restored image result one-fourth the size of the original. Consequently, a direct visual comparison between the two may favor the captured image due to their size disparity. This discrepancy, stemming from differing dimensions and pixel counts, hinders direct comparability. 

In the image restoration outcomes, the study observed that the selection of background image parameters, involving human intervention, influences the evaluation metrics of the restored images. To mitigate this influence, a strategy of multiple selections to obtain an averaged result was employed, aiming for more accurate evaluation outcomes. To compare and quantify the quality of image enhancement under non-reference conditions across various turbidity levels, multiple evaluation metrics were employed during testing, as presented in Table 1. These metrics include entropy [25], EME [26], and Sobel operator [27]. Entropy gauges the amount of information contained in the enhanced image, EME indicates the degree of local grayscale variation in the image enhancement result, and the Sobel operator assesses edge clarity. These metrics yield higher values for superior image enhancement results. We tested image enhancement outcomes under varying turbidity conditions by gradually adding 200 mL of milk to the pool three times, totaling 600 mL. The milk composition included 2.6 g/100 mL of protein and 4.7 g/100 mL of fat. Based on the computational outcomes, the data in Table 1 below was formulated.

According to the data provided in the table and the image results shown above, it is evident that the algorithm proposed in this paper can effectively enhance photos taken in relatively turbid environments. The EME values obtained by our algorithm are lower than those of the PD method, and the entropy values are higher, approximately twice that of the PD method. Therefore, our method processes images with more information, resulting in clearer images. The variation of the EME indicator suggests that as turbidity increases, the efficiency of image enhancement begins to decrease. This phenomenon arises from the fact that although the quality of the enhanced image is improved compared to the original image, the initial capture results of the sensor are not sufficient to recover a clear image. Therefore, the final restoration results under high turbidity cannot be practically applied to underwater visual systems. Hence, a comprehensive approach is needed to assess image clarity results, and the actual enhanced images should be considered as the reference.

**Figure 8:**
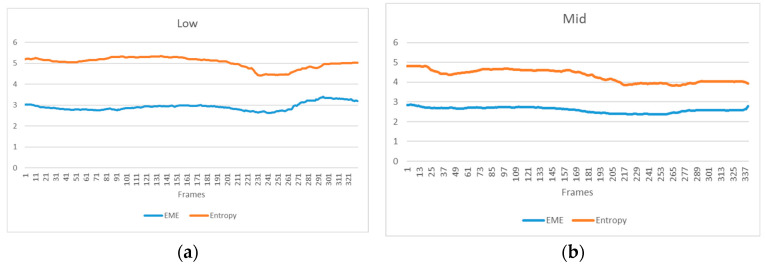
This is a figure. Schemes follow another format. If there are multiple panels, they should be listed as: (**a**) video sequence evaluation indicators in low concentration and (**b**) video sequence evaluation indicators in mid concentration.

Additionally, we validated the image processing method for video sequences proposed in Section 2.4. As per the algorithm’s principle, the environmental parameters for the first frame image are computed. Subsequently, these parameters are employed to calculate and derive processing results for subsequent frames. When the computed results from the parameters do not meet the current requirements, the system will reutilize the current frame to calculate the parameters and complete the remaining processing steps. A comprehensive evaluation of each frame was conducted through the amalgamation of EME and ENT metrics, forming distinct variations in images for different concentrations. The variation graph for low concentration is illustrated in Figure 8a, and for moderate concentration, it is shown in Figure 8b. Analyzing the variations in the two images, it can be inferred that when environmental variables such as light intensity and turbidity, which can affect imaging parameter estimation, remain relatively stable, the proposed video enhancement method offers a convenient and minimally intervention-prone means of stable imaging.

## 4. Conclusions and Outlook

We have successfully developed an underwater image and video enhancement method using calculations based on Stokes linear polarized light. By estimating the scattered light intensity in the background light region, the integration of Stokes linear polarized light with the underwater polarized light imaging model has enhanced the traditional linear polarized light underwater imaging model, leading to significant suppression of scattering effects. Moreover, considering the underwater working environment, a video imaging system requiring minimal human–machine interaction has been devised. This system utilizes parameters calculated from the first frame for subsequent frame computations, and it employs constraint-based evaluation metrics. Parameters are recalculated when the evaluation metrics are not met, thereby achieving target requirements. Compared to other research in this domain, we are the first to apply the polarization enhancement algorithm to video sequences, yielding more stable results and providing new insights for underwater visual applications. In the medium to low turbidity conditions, stable recovery results have been achieved. This method effectively addresses video sequences under certain conditions, freeing itself from the constraints of traditional frame-by-frame processing in static models. However, its robustness weakens when interference factors like ambient light cause significant variations in parameter estimation. The experimental results underscore that this method excels in slowly changing lighting environments with relatively stable turbidity conditions. It adeptly processes video sequences and yields significantly clearer restored sequences.

Throughout this study, opportunities for enhancing and innovating this model have emerged. Firstly, in terms of polarized light directions, enriching the optical information that can be processed within the proposed system could involve incorporating more polarized light information, including circularly polarized light calculations. Secondly, in the realm of physical imaging models, some prior models have been identified that operate without the need for background regions and priors. Integrating these methods into the system proposed in this study could reduce human–machine interaction and conserve computational resources. Lastly, the realm of DL offers a potential avenue for improvement. Incorporating neural network operations in image input and processing could aid in lower computational complexity and enhanced object resolution. This approach would significantly contribute to image clarity restoration. This study envisions that this method and its various potential improvements will effectively assist underwater robots in performing tasks in turbid environments and find applications in other areas of scattering suppression imaging.

## Figures and Tables

**Table 1:** Evaluate results.

Method	Turbidity Level	Evaluation Criteria		
		EME	Entropy	Sobel
Our method	Low	7.341	4.975	17.274
	Mid	5.436	5.192	13.065
	High	5.631	4.983	13.198
PD method	Low	9.454	2.409	16.148
	Mid	7.101	3.091	18.368
	High	8.384	2.876	18.977

## Data Availability

The data underlying the results presented in this paper are currently not publicly available as they involve projects funded by private sources. However, access to the data can be negotiated with the authors based on specific needs.
